# Hyperbaric oxygen treatment and toll-like receptor 5 in radioprotection of epithelial cells

**DOI:** 10.1371/journal.pone.0356247

**Published:** 2026-08-14

**Authors:** Lana Sallam, Lucie Rychlíková, Johan Mölne, Martin O. Bergo, Åsa Torinsson Naluai, Helene Seeman-Lodding, Nicklas Oscarsson, Daniel Giglio

**Affiliations:** 1 Department of Oncology, Institute of Clinical Sciences, Sahlgrenska Academy at the University of Gothenburg, Gothenburg, Sweden; 2 Department of Pharmacology, Institute of Neuroscience and Physiology, Sahlgrenska Academy at the University of Gothenburg, Gothenburg, Sweden; 3 Department of Pathology, Sahlgrenska University Hospital, Gothenburg, Sweden; 4 Department of Medicine, Huddinge, Karolinska Institutet, Stockholm, Sweden; 5 Department of Laboratory Medicine and Core Facilities, Institute of Biomedicine, Sahlgrenska Academy at the University of Gothenburg, Gothenburg, Sweden; 6 Department of Anesthesiology and Intensive Care Medicine, Sahlgrenska University Hospital, Gothenburg, Sweden; 7 Department of Anesthesiology and Intensive Care Medicine Institute of Clinical Sciences, Sahlgrenska Academy at the University of Gothenburg, Gothenburg, Sweden; 8 Department of Medicine and Oncology, Southern Älvsborg Hospital, Borås, Sweden; Manipal Academy of Higher Education School of Life Sciences, INDIA

## Abstract

**Background:**

Irradiation of tumours in the pelvic area may lead to adverse effects such as vaginal fibrosis and dyspareunia. We investigated the radioprotective effects of hyperbaric oxygen (HBOT) and toll-like receptor 5 (TLR5) in epithelial cells.

**Methods and materials:**

Endothelial cells (HUVEC), urothelial cells (UROtsa), and cervical epithelial cells (HeLa) were irradiated with 6 Gy and subsequently underwent HBOT. Cells were counted and qPCR was performed on HeLa for the expressions of TLR5, downstream signalling molecules, oxidative stress, and cytokines. The effects of the TLR5 agonist flagellin and the TLR5 antagonist TH1020 on radiation-induced cell death and oxidative stress were assessed.

**Results:**

HBOT attenuated radiation-induced epithelial cell death. In HeLa, radiation reduced TLR5, TRIF, NRF2, HIF-α and catalase expression. HBOT reversed their expression. The TLR5 agonist flagellin protected against radiation-induced cell death in HeLa and the TLR5 antagonist TH1020 inhibited the radioprotective effects by flagellin.

**Conclusions:**

HBOT antagonizes radiation-induced cell death and normalizes the expressions of antioxidants, TLR5 and connected downstream signalling transcripts. TLR5 signalling contributes to protection from radiation-induced epithelial cell death, which supports further exploration of combined HBOT-TLR5-targeted strategies for mucosal radioprotection.

## Introduction

Damage-associated molecular patterns (DAMPs) and pathogen-associated molecular patterns (PAMPs) from bacteria and viruses may trigger an immune response by activating toll-like receptors (TLRs) on immune cells and epithelia of different tissues including the uterine cervix [1]. Activation of TLRs triggers downstream signalling through the adaptor molecules myeloid differentiation primary response 88 (MyD88) and TIR-domain-containing adapter-inducing interferon-β (TRIF), leading to cytokine release via the activation of transcription factors such as NF-κB [2]. TLRs may also be activated by other stimuli than bacteria and viruses, *i.e.*, from ionizing radiation and ultraviolet radiation [3]. TLR5 activation has been reported to induce radioprotection but the underlying mechanism remains incompletely understood. For example, the administration of the TLR5 agonist flagellin protected mice exposed to acute radiation-induced tissue injury and resulted in improved survival [4]. Wang et al reported that the TLR5 agonist CBLB502 protected mice against radiation-induced intestinal injury by reversing the expression of radiation-induced genes and regulating immune processes and metabolic pathways [5].

Patients treated with radiotherapy due to tumours of the pelvic area, such as of the cervix and the prostate gland, may experience adverse effects including radiation cystitis, radiation proctitis, vaginal fibrosis and dyspareunia [6,7]. We have previously shown that hyperbaric oxygen (HBOT) is an important treatment modality against radiation cystitis leading to durable clinical efficacy [8,9]. The underlying pharmacodynamics of HBOT against radiation-induced side effects of the genitourinary tract has been studied only sparsely. In an animal model of radiation cystitis, we demonstrated that HBOT attenuated radiation-induced oxidative stress and normalised the expression of antioxidants and cytokines in the urinary bladder [10]. Further, in another animal model of radiation cervicitis, we showed that radiation induced immunosuppression, oxidative stress and an upregulation of TLR5 14 days following cervical irradiation [11].

Studies by us and others show that HBOT can reverse radiation-induced alterations in inflammatory and antioxidant pathways. Additionally, TLR5 has been implicated in radioprotection in animal models. In the present study, we further investigated the pathophysiological responses of epithelial cells to radiation and examined the effects of HBOT on radiation-induced changes in cell survival, antioxidation, TLR and downstream TLR adaptor proteins, and inflammatory signalling. Furthermore, we evaluated the radioprotective role of TLR5 specifically in cervical epithelial cells.

## Materials and methods

### Cell culture, irradiation and HBOT

HUVEC was cultured in endothelial cell growth medium (EBM-2, ref no cc3156; Fisher Scientific, Gothenburg, Sweden) supplemented with the EGM-2 MV BulletKit (cc4147; Lonza, Walkersville, USA), supplemented with fetal bovine serum (FBS, 10%; Sigma-Aldrich, Stockholm, Sweden) and penicillin-streptomycin (PEST 1%, Sigma-Aldrich). UROtsa and HeLa were cultured in Dulbecco’s Modified Eagle’s medium-low glucose (DMEM, Sigma-Aldrich), supplemented with FBS (10%) and PEST (1%). Cells were incubated at 37°C in humidified conditions at 5% CO_2_, and were passaged every 3–7 days before reaching confluence. Culture media was changed three times a week.

In the first experiment, HUVEC, UROtsa and HeLa cells, cultured in 96-well plates, were irradiated 2–20 Gy at a dose rate of 1,65 Gy/min (RS 2000 X-ray Biological Irradiator, Rad Source Technologies, Suwanee, GA, USA). In the second experiment, cells were seeded at a density of 10,000 cells per well in 96-well plates. Cells were either irradiated with 6 Gy or placed outside the incubator at room temperature and then placed back in the cell incubator. Irradiated cells and non-irradiated cells were either exposed to HBOT (HBOT-rad and HBOT-ctrl, respectively) or placed outside the incubator at room temperature (Sham-rad and Sham-ctrl, respectively). Each group consisted of n = 11–12 wells.

During HBOT, the cells were placed in a hyperbaric chamber (GDA, Gothenburg, Sweden) and exposed to 200 kPa for 90 minutes, conditions shown to be effective against radiation-induced side effects in animal models and clinical practice [10,12]. HBOT was performed 6 h after irradiation for HeLa and UROtsa. For HUVEC, exposing irradiated cells 6 h later with HBOT induced an increase in cell death compared with sham-treated irradiated cells. Therefore, another protocol for HUVEC was employed where HUVEC cells were exposed to HBOT twice (3 h and 10 h post-irradiation). The rationale for a seven-hour time window between sessions of HBOT was for the cells to recover after being outside of the incubator. No changes of media occurred after irradiation/control treatment. HeLa, UROtsa and HUVEC cells were counted under the microscope in a blinded manner 24 hours after irradiation. After irradiation/control treatment and HBOT/sham treatment, HeLa cells were washed twice with PBS and 50 μL of bovine serum albumin (BSA) 1 mg/ml was added. The samples were then stored at −80°C.

### TLR5 experiments

One hour before irradiation, the TLR5 agonist flagellin (100 ng/ml), the TLR5 antagonist TH1020 (10 µM) or the combination of flagellin (100 ng/ml) and TH1020 (10 µM) was added to the HeLa cells (n = 12). When cells were treated with both TH1020 and flagellin, TH1020 was administered first followed by flagellin 30 minutes later 30 minutes before irradiation. Control cells were kept outside the incubator while the experimental groups received treatment. HeLa cells were grown for 24 hours before counting the cells in a blindly manner under the microscope. Subsequently, the cells were washed twice with PBS and 50 μL of BSA 1 mg/ml was added. The samples were then stored at −80°C.

### Live-dead staining

HeLa cells were cultured in 12-well chamber slides and kept in the incubator until reaching confluence. Cells were either irradiated with 6 Gy (n = 8), irradiated with 6 Gy, followed by HBOT 6 hours later using the same protocol as above (n = 8) or received no treatment (control cells; n = 8). The LIVE/DEAD™ Viability/Cytotoxicity Kit for mammalian cells (Thermo Fisher Scientific, MA, USA; L3224) was used. The staining solution was prepared by adding 5 μL calcein AM and 20 μL ethidium homodimer-1 to 10 mL PBS. At 24 h after irradiation the cell medium was removed and 200 μL of the staining solution was added directly to the cells. The cells were incubated for 30 min at 25°C. Following incubation, the staining solution was removed, a coverslip was mounted on top of the cells, and cells were imaged using the Nikon Eclipse 90i (Nikon, Minato, Japan). Identical lookup tables (LUTs) settings were applied to all images. The total number of cells and the number of dead cells were counted in each image, and the percentage of dead cells was calculated.

### Cell lysis and qPCR

Direct cell lysis in 1 mg/ml BSA was performed, which maximizes the lysis process while preserving mRNA as previously described [13]. Complementary DNA (cDNA) was synthesized from the cell samples using the High-capacity cDNA reverse transcription kit (cat. No. 4368813, Thermo Fisher Scientific). Quantitative polymerase chain reaction (qPCR) was performed using TaqMan^®^ technology (Thermo Fisher Scientific). The reaction mixture consisted of gene primers, sample cDNA and master mix (2x universal TaqMan Mastermix, Thermo Fisher Scientific). All primer-sample reactions were run in triplicate in 384-well plates on a QuantStudio 12K Flex Sequence Detection System (Thermo Fisher Scientific). Target genes were aryl hydrocarbon receptor nuclear translocator (ARNT/HIF-1β), caspase 3, catalase, heme oxygenase 1 (HO1), hypoxia inducible factor 1 subunit alpha (HIF-1α), interleukin 1 receptor associated kinase 1 (IRAK1), interleukin 1B, interleukin 6, Kelch-like ECH-associated protein 1 (KEAP1), MYD88, NF-κB p65 subunit (RELA), Nuclear factor erythroid 2-related factor 2 (NRF2), nuclear factor kappa B subunit 1 (NF-κB1), superoxide dismutase 1 (SOD1), superoxide dismutase 2 (SOD2), transforming growth factor (TGF) beta1, tumor necrosis factor (TNF), TRIF, TLR5. Reference genes were glucuronidase beta (GUSB), pumilio RNA binding family member 1 (PUM1), glyceraldehyde-3-phosphate dehydrogenase (GAPDH) and actin beta (ACTB). List of primers is displayed in S1 Table. The fold change was calculated using the 2- ΔΔC method where each target gene was normalized to ACTB or the mean of GUSB and ACTB.

### Statistical analysis

Statistical significance for unpaired data was determined with the Mann-Whitney test. The Kruskal-Wallis test followed by the Dunn’s multiple comparisons test was performed when multiple comparisons were made. A p-value of less than 0.05 was considered statistically significant. Data was presented as mean ± standard error of the mean (SEM). Graphs were made and parameters calculated using GraphPad Prism 10.5.0 (GraphPad Software, Inc., San Diego, USA).

## Results

**Fig 1:**
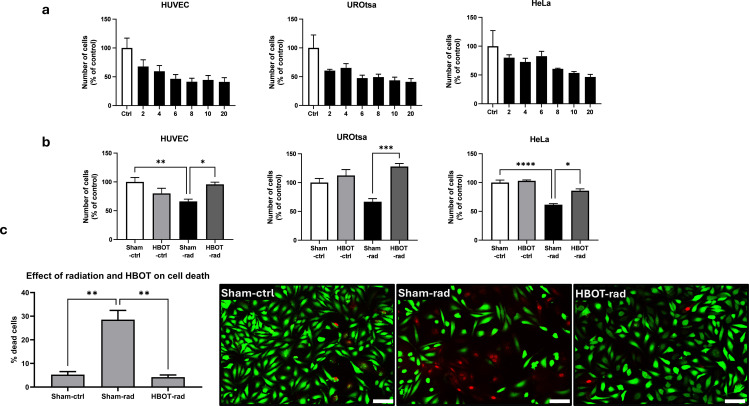
The average number of HUVEC (n = 7 for Ctrl, 2-8 Gy and n = 7 for 10-20 Gy), UROtsa (n = 4 for Ctrl, 2-8 Gy and n = 3 for 10-20 Gy) and HeLa cells (n = 3 for Ctrl, 2-8 Gy and n = 2 for 10-20 Gy) 24 h after irradiation (2-20 Gy)/control treatment (n = 2-7; a). The average number of control cells (Sham-ctrl), after HBOT of control cells (HBOT-ctrl), after irradiation (Sham-rad) and after irradiation followed by HBOT (HBOT-rad; n = 6-12; b). The precentage of dead cells in control HeLa cells (Sham-ctrl; n = 8), after irradiation (Sham-rad; n = 8) and after irradiation followed by HBOT (HBOT-rad; n = 8). Representative microphotographs are shown to the right where living cells are coloured green and dead cells are coloured red (magnification x100; the white horizontal bar indicates 10 µm; c). Lookup tables (LUTs) were adjusted for each fluorescence channel to improve visualization of dead cells. Multiple comparisons were analyzed using the Kruskal-Wallis test followed by Dunn’s multiple comparisons test.Vertical bars indicate SEM. * indicates p < 0.05, ** indicates p < 0.01, *** indicates p < 0.001 and **** indicates p < 0.0001.

Radiation induced cell death in the endothelial cell line HUVEC, the urothelial cell line UROtsa and the cervical epithelial cell line HeLa in a dose-dependent manner (Fig 1a). One session of HBOT (6 h post-radiation) in HeLa and UROtsa and two sessions of HBOT in HUVEC (3 h and 10 h post-radiation) attenuated cell death 24 h post-radiation (Fig 1b-c).

**Fig 2:**
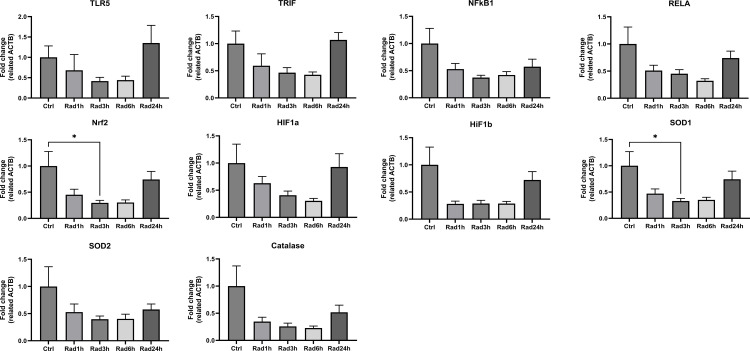
The average mRNA expressions of TLR5, TRIF, NF-κB1, RELA, NRF2, HIF1-α, HIF1-β, SOD1, SOD2 and catalase in control HeLa cells (Ctrl) and 1-24 h after irradiation (Rad1h-Rad24h; n = 12). Multiple comparisons were analyzed using the Kruskal-Wallis test followed by Dunn’s multiple comparisons test. Vertical bars indicate standard error of the mean (SEM). * indicates p < 0.05.

Radiation of HeLa induced time-dependent downregulations of several genes up to 6 hours. These genes included TLR5 and down-stream signalling pathways (TRIF, NF-κB1, RELA), NRF2, HIF1-α and HIF1-β and antioxidants (SOD1, SOD2 and catalase; Fig 2).

**Fig 3:**
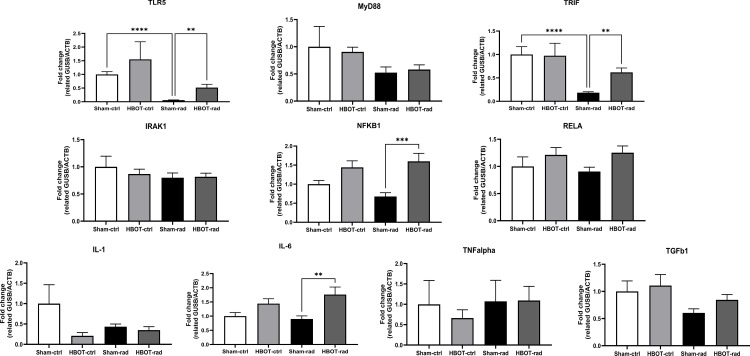
The average mRNA expressions of TLR5, MyD88, TRIF, IRAK1, NF-κB1, RELA, IL-1, IL-6, TNFα and TGFβ1 in HeLa of control cells (Sham-ctrl; n = 12), after HBOT of control cells (HBOT-ctrl; n = 12), after irradiation (Sham-rad; n = 12) and after irradiation followed by HBOT (HBOT-rad; n = 11-12). **Multiple comparisons were analyzed using the Kruskal-Wallis test followed by Dunn’s multiple comparisons test.** Vertical bars indicate standard error of the mean (SEM). ** indicates p < 0.01, *** indicates p < 0.001 and **** indicates p < 0.0001.

**Fig 4:**
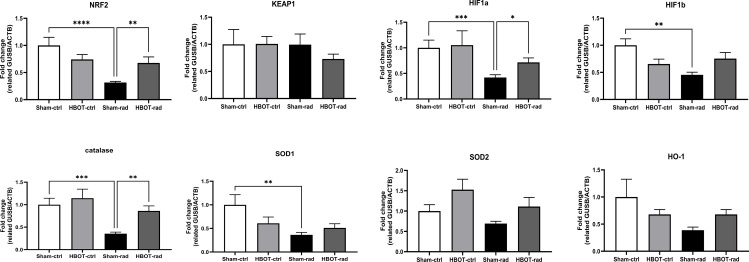
The average mRNA expressions of NRF2, KEAP1, HIF1-α, HIF1-β, catalase, SOD1, SOD2, and HO-1 in HeLa in control cells (Sham-ctrl; n = 12), after HBOT of control cells (HBOT-ctrl; n = 12), after irradiation (Sham-rad; n = 12) and after irradiation followed by HBOT (HBOT-rad; n = 12). Multiple comparisons were analyzed using the Kruskal-Wallis test followed by Dunn’s multiple comparisons test. Vertical bars indicate standard error of the mean (SEM). * indicates p < 0.05, ** indicates p < 0.01, *** indicates p < 0.001 and **** indicates p < 0.0001.

Radiation-induced down-regulations of TLR5, TRIF, NF-κB1, NRF2, HIF1-α and catalase were reversed by HBOT (p < 0.001–0.05) and tended to be reversed by HBOT for HIF1-β (Fig 3 and Fig 4).

**Fig 5:**
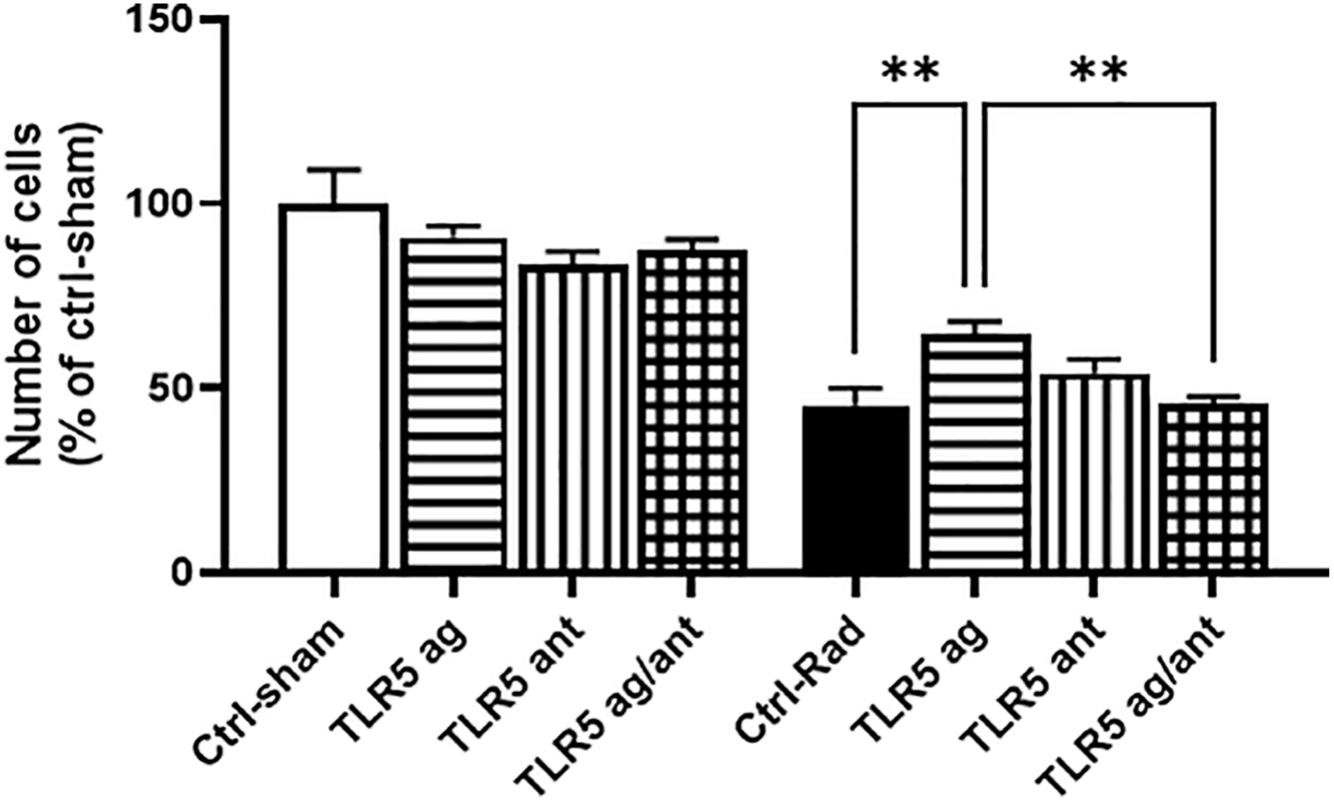
The average number of HeLa cells of control cells (Ctrl) and irradiated cells (Rad) in the presence of flagellin (100 ng/ml; TLR5 agonist), in the presence of TH1020 (10 µM, TLR5 antagonist) and in the presence of flagellin (100 ng/ml; TLR5 agonist) and TH1020 (10 µM; TLR5 antagonist) 24 h after irradiation (n = 12). Multiple comparisons were analyzed using the Kruskal-Wallis test followed by Dunn’s multiple comparisons test. Vertical bars indicate standard error of the mean (SEM). ** indicates p < 0.01.

The TLR5 agonist flagellin (100 ng/ml) and the TLR5 antagonist TH1020 (10 µM) had no effects on control HeLa cells (Fig 5). In contrast, flagellin (100 ng/ml) attenuated radiation-induced cell death (p < 0.01) in HeLa cells, while TH1020 (10 µM) had no effect *per se* on cell radiation-induced cell death. The protective effect of flagellin (100 ng/ml) against radiation-induced cell death was blocked in the presence of TH1020 (10 µM; p < 0.01; Fig 5).

6 and 24 hours of incubation with flagellin (100 ng/ml) and TH1020 (10 µM) had no effect on the expression of TLR5, MyD88, TRIF, NRF2, HIF1-α, HIF1-β, SOD1, SOD2, catalase or caspase-3. In the presence of both flagellin (100 ng/ml) and TH1020 (10 µM), the expression of TLR5 was increased compared with only flagellin (100 ng/ml) at 24 hours in irradiated cells (p < 0.05), while the expression of TRIF was increased compared with only flagellin (100 ng/ml) in irradiated cells at 6 h (p < 0.05) and tended to do so also at 24 h (p = 0.053; Fig S1; n = 12 in each group).

## Discussion

The present study showed that HBOT may block radiation-induced cell death and affect radiation-induced changes in oxygenation, antioxidation and inflammation. Moreover, TLR5 activation led to radioprotection in cervical epithelial cells.

To evaluate the pharmacodynamics of HBOT and the possible involvement of TLR5 in radioprotection, we studied the effects of HBOT and TLR5 in irradiated cells derived from the endothelium, the urothelium, and the cervical epithelium. Radiation-induced cell death in HUVEC, UROtsa and HeLa occurred already at a dose of 2 Gy, which is in line with previous studies showing that oxidative stress and cell death in HUVEC, UROtsa and HeLa occur already at doses below 2 Gy [14–17].

We demonstrate that HBOT may reduce radiation-induced cell death, consistent with earlier studies showing that the number of apoptotic intestinal epithelial cells decreases in irradiated mice treated with HBOT [18]. One session of HBOT at 200 kPa for 90 min was able to attenuate radiation-induced cell death of UROtsa and HeLa and two sessions of HBOT for HUVEC. In our pilot experiments, we observed that a 7 h interval between HBOT sessions was required to attenuate cell death induced by radiation in HUVEC, while shorter intervals resulted in further cell death (data not shown). We speculate that, since HBOT also induces increased levels of reactive oxygen species (ROS), the total load of ROS might be too high when repeated HBOT is applied at short interval [19]. This is further supported by findings by Yuan et. al. showing that DNA damage occurs shortly after HUVEC exposure to HBOT [20]. HBOT normalized radiation-induced changes in antioxidant expression, possibly through the NRF2 signalling pathway. HBOT has been shown to normalize NRF2 expression in the diabetic foot [21]. Evidence from animal models of spinal cord injury [22], graft-versus-host disease [23], and myocardial infarction [24] highlights the pivotal role of NRF2 in determining the clinical efficacy of HBOT.

NRF2 binds antioxidant response element (ARE) and promotes the transcription of multiple downstream antioxidant genes including HO-1, SODs, and catalase [25]. We could not observe any change in inflammation nor in oxidative stress or cell death in control HeLa cells exposed to HBOT. These findings are consistent with clinical observations that repeated HBOT does not induce systemic oxidative stress or inflammation in healthy volunteers [26]. In contrast, oxidative stress reflected by reductions in the antioxidants catalase and SOD1 and in NRF2 and HIF1-α occurred in irradiated HeLa cells.

TLR5 and the downstream mediator TRIF were reduced 24 h after radiation. The expressions of TLR5, TRIF and NF-κB1 in irradiated cells were increased after HBOT. We have previously showed that TLR5 and TRIF are altered in the same direction in response to radiation, suggesting that TRIF may act as an adaptor molecule for TLR5 in the cervical epithelium [11]. The activation of NF-κB pathway promotes cell survival by triggering genes that regulate apoptosis and antioxidation [27]. Therefore, we further explored whether TLR5 has radioprotective effects by stimulating HeLa cells with the TLR5 agonist flagellin in the presence and absence of the TLR5 antagonist TH1020. The presence of flagellin protected HeLa cells against radiation-induced cell death. While TH1020 *per se* did not have any effects on HeLa, TH1020 inhibited the radioprotective effects of flagellin.

Animal models report that TLR5-induced radioprotection goes via NF-κB and up-regulation of SOD-2 [4]. Moreover, TLR5-dependent radioprotection of the murine testes involves NF-κB activation and increased Bcl-2 expression [28]. We did, however, not observe any changes in the expressions of downstream MyD88, TRIF or antioxidant genes following flagellin/TH1020 treatment after radiation. The mechanisms behind flagellin-induced radioprotection may potentially involve TLR5-dependent and TLR5-independent downstream intracellular pathways. To note, besides activating TLR5, flagellin may activate the NAIP5-NLRC4 inflammasome and caspase-1 [29]. Caspase-1 induces TRIF cleavage thereby regulating authophagy and β-interferon production of macrophages [30]. Speculatively, a downstream interaction between TLR5 and NAIP5-NLRC4 pathways may occur giving the unexpected upregulations of TLR5 and TRIF we currently observed.

Our study has several limitations. First, we investigated radioprotection in cancer cell lines as a surrogate model for healthy tissue. Second, we could not provide evidence for a causal interaction between the radioprotective mechanisms of HBOT and TLR5 even though HBOT normalized mRNA levels of TLR5 after irradiation. Other antioxidant or intracellular signalling pathways, beyond those examined here, may contribute to TLR5-mediated radioprotection. Third, radiation-induced oxidative stress was currently only indirectly assessed by assessing levels of antioxidants and the cell count rather than by direct quantification of oxidative damage.

In conclusion, TLR5 activation occurs in cervical epithelial cells in response to radiation, which may induce protection against radiation-induced cell death. HBOT attenuates radiation-induced cell death and reverses radiation-induced changes in oxidative stress and oxygenation. HBOT or treatments targeting TLR5 may potentially be used to prevent or attenuate adverse effects of radiotherapy against the genital tract.

## Supporting information

S1 Fig Average mRNA expressions of immune, hypoxia, oxidative stress, and apoptosis-related genes in the absence and presence of TLR5 agonist and/or antagonist. (TIF)

S1 Table mRNA primer list. (DOCX)

S1 File Raw data. (XLSX)
