# Supplementary material for: Hyperbaric oxygen treatment and toll-like receptor 5 in radioprotection of epithelial cells
Source: PLoS One. 2026 Aug 14;21(8):e0356247. doi: 10.1371/journal.pone.0356247 (PMC13475910; doi:10.1371/journal.pone.0356247)
Supplement: Supplementary file 1 [file pone.0356247.s006.pdf]

Gothenburg, Sweden, 28 May 2026

Dear Editor,

Thank you for the opportunity to revise and resubmit our manuscript. We have carefully addressed all comments and suggestions. No further changes have been made in the manuscript.

Daniel Giglio

MD, PhD, Associate Professor

Response to reviewers´comments

Reviewer #1: why the sample size is 12 in the supplemantary data ?it need so many repulation ?please explain it

Answer: When we made multiple comparisons with Kruskal-Wallis for qPCR data we chose to have n=12 to have a sufficient number of samples to compensate for variations between samples.

Reviewer #2:

1. Sallam et al. and co-authors have suggested a mechanistic role of HBOT in minimizing radiation-induced cellular toxicity and downstream inflammatory and oxidative pathways, but they focused only on the transcriptional regulation. In contrast to current study, Jia Liu et al. 2022 (PMID: 36495316) earlier demonstrated that HBOT co-exposure with radiation significantly strengthened cytotoxicity via ferroptotic cell death in oral squamous cell carcinoma cells. At this stage, it's highly recommended to confirm this mechanism at protein level as well. Radiation induced toxicity and NRF2 mediated regulation of oxidative stress need to be confirmed by detecting ROS, 4-HNE or MDA.

Answer: In the present study we assessed radiotherapy-induced toxicity and effects of HBO2 on several different important biological pathways primarily on the mRNA level. We have in Discussion in the section covering limitations highlighted that oxidative stress was only measured indirectly by measuring antioxidants.

2. Sallam et al. and co-authors have demonstrated that HBOT regulates TLR5 connected downstream signaling. While Rinaldi et al. 2011 and Kang et al. 2015 (PMID: 21567111 and 25973000) have highlighted the link between HBOT and other TLRs (TLR2 and TLR4), it's also advisable to check the levels of other TLRs in connection with radiation.

Answer: We acknowledge that other TLRs may be affected by irradiation as well. We have for example observed that radiation may downregulate TLR4 in the rat urinary bladder (1). However, based on our previous animal model of radiation-induced cervicitis, we observed only that TLR5 increased in expression among all TLRs (2). Moreover, the role of TLR5 has also been demonstrated in other models of radiation-induced radiotoxicity (3, 4). The article referred to by Reviewer 2 by Rinaldi et. al. (2011) describes down-regulation of TLR2 and TLR4 not in radiation-induced toxicity but in zymosan-induced general inflammation and the effects by HBO2 (5). The other article referred to by Reviewer 2 by Kang et. al. (2015) deals with spinal cord injury (and not radiation) and activation of TLR4 and effects of HBO2 (6).

References

1. Giglio D, Wasen C, Molne J, Suchy D, Swanpalmer J, Jabonero Valbuena J, et al. Downregulation of toll-like receptor 4 and IL-6 following irradiation of the rat urinary bladder. Clin Exp Pharmacol Physiol. 2016;43(7):698-705.
2. Mukanyangezi MF, Podmolikova L, Al Hydad W, Tobin G, Giglio D. Radiation induces changes in toll-like receptors of the uterine cervix of the rat. PLoS One. 2019;14(4):e0215250.
3. Burdelya LG, Krivokrysenko VI, Tallant TC, Strom E, Gleiberman AS, Gupta D, et al. An agonist of toll-like receptor 5 has radioprotective activity in mouse and primate models. Science. 2008;320(5873):226-30.
4. Wang Q, Duan J, Hong J, Ding K, Tai F, Zhu J, et al. Toll-like Receptor Agonist CBLB502 Protects Against Radiation-induced Intestinal Injury in Mice. In Vivo. 2024;38(4):1636-48.
5. Rinaldi B, Cuzzocrea S, Donniacuo M, Capuano A, Di Palma D, Imperatore F, et al. Hyperbaric oxygen therapy reduces the toll-like receptor signaling pathway in multiple organ failures. Intensive Care Med. 2011;37(7):1110-9.
6. Kang N, Hai Y, Yang J, Liang F, Gao CJ. Hyperbaric oxygen intervention reduces secondary spinal cord injury in rats via regulation of HMGB1/TLR4/NF-kappaB signaling pathway. Int J Clin Exp Pathol. 2015;8(2):1141-53.
