# Supplementary material for: Hyperbaric oxygen treatment and toll-like receptor 5 in radioprotection of epithelial cells
Source: PLoS One. 2026 Aug 14;21(8):e0356247. doi: 10.1371/journal.pone.0356247 (PMC13475910; doi:10.1371/journal.pone.0356247)
Supplement: Supplementary file 2 [file pone.0356247.s002.docx]

| **Target name** | **Assay ID (Thermo Fisher Scientific)** |
| --- | --- |
| GUSB | Hs00939627_m1 |
| GAPDH | Hs99999905_m1 |
| PUM1 | Hs00472881_m1 |
| TLR5 | Hs01920773_s1 |
| Ticam1 | Hs00706140_s1 |
| NFkB1 | Hs00765730_m1 |
| RELA | Hs01042014_m1 |
| NFE2L2 | Hs00975960_m1 |
| HIF1A | Hs00153153_m1 |
| ARNT | Hs01121918_m1 |
| SOD1 | Hs00533490_m1 |
| SOD2 | Hs00167309_m1 |
| CAT | Hs00156308_m1 |
| MyD88 | Hs00182082_m1 |
| IRAK1 | Hs00155570_m1 |
| IL1B | Hs01555410_m1 |
| IL6 | Hs00174131_m1 |
| TNF | Hs00174128_m1 |
| TGFB1 | Hs00998133_m1 |
| KEAP1 | Hs00202227_m1 |
| HMOX1 | Hs01110250_m1 |
| ACTB | Hs01064291_g1 |
| CASP3 | Hs00234387_m1 |

Table S1. Primer list.
