# Supplementary material for: Hyperbaric oxygen treatment and toll-like receptor 5 in radioprotection of epithelial cells
Source: PLoS One. 2026 Aug 14;21(8):e0356247. doi: 10.1371/journal.pone.0356247 (PMC13475910; doi:10.1371/journal.pone.0356247)
Supplement: Peer review history

# Peer review: Decision Letter 0

**DOI:** 10.1371/journal.pone.0356247.r001

**Authors:** Budheswar Dehury

26 Dec 2025

Dear Dr. Giglio,

Thank you for submitting your manuscript to PLOS ONE. After careful consideration, we feel that it has merit but does not fully meet PLOS ONE’s publication criteria as it currently stands. Therefore, we invite you to submit a revised version of the manuscript that addresses the points raised during the review process.

==============================

The MS has been reviewed by the Editorial member as well as reviewer, all opined their view for a major revision before it can be considered further. Carefully revise the MS and address the reviewer and editorial comments.

==============================

Please submit your revised manuscript by Feb 09 2026 11:59PM. If you will need more time than this to complete your revisions, please reply to this message or contact the journal office at plosone@plos.org. When you're ready to submit your revision, log on to https://www.editorialmanager.com/pone/ and select the 'Submissions Needing Revision' folder to locate your manuscript file.

- A letter that responds to each point raised by the academic editor and reviewer(s). You should upload this letter as a separate file labeled 'Response to Reviewers'.
- A marked-up copy of your manuscript that highlights changes made to the original version. You should upload this as a separate file labeled 'Revised Manuscript with Track Changes'.
- An unmarked version of your revised paper without tracked changes. You should upload this as a separate file labeled 'Manuscript'.

If you would like to make changes to your financial disclosure, please include your updated statement in your cover letter. Guidelines for resubmitting your figure files are available below the reviewer comments at the end of this letter.

If applicable, we recommend that you deposit your laboratory protocols in protocols.io to enhance the reproducibility of your results. Protocols.io assigns your protocol its own identifier (DOI) so that it can be cited independently in the future. For instructions see: https://journals.plos.org/plosone/s/submission-guidelines#loc-laboratory-protocols. Additionally, PLOS ONE offers an option for publishing peer-reviewed Lab Protocol articles, which describe protocols hosted on protocols.io. Read more information on sharing protocols at https://plos.org/protocols?utm_medium=editorial-email&utm_source=authorletters&utm_campaign=protocols.

We look forward to receiving your revised manuscript.

Kind regards,

Budheswar Dehury

Academic Editor

PLOS One

Journal Requirements:

When submitting your revision, we need you to address these additional requirements.

1. Please ensure that your manuscript meets PLOS ONE's style requirements, including those for file naming. The PLOS ONE style templates can be found at

https://journals.plos.org/plosone/s/file?id=wjVg/PLOSOne_formatting_sample_main_body.pdf and

https://journals.plos.org/plosone/s/file?id=ba62/PLOSOne_formatting_sample_title_authors_affiliations.pdf

2. Thank you for stating the following financial disclosure:

“The present project was supported by the King Gustav V Jubilee Clinical Cancer Research Foundation, Gothenburg, Sweden, and the Western County Council Clinical Research Foundation [VGFOUREG-374501], Sahlgrenska Academy, Gothenburg, Sweden.”

Please state what role the funders took in the study.  If the funders had no role, please state: "The funders had no role in study design, data collection and analysis, decision to publish, or preparation of the manuscript."

If this statement is not correct you must amend it as needed.

Please include this amended Role of Funder statement in your cover letter; we will change the online submission form on your behalf.

3. Thank you for stating the following in the Acknowledgments Section of your manuscript:

“The present project was supported by the King Gustav V Jubilee Clinical Cancer Research Foundation, Gothenburg, Sweden, and the Western County Council Clinical Research Foundation [VGFOUREG-374501], Sahlgrenska Academy, Gothenburg, Sweden.”

We note that you have provided additional information within the Acknowledgements Section that is not currently declared in your Funding Statement. Please note that funding information should not appear in the Acknowledgments section or other areas of your manuscript. We will only publish funding information present in the Funding Statement section of the online submission form.

Please remove any funding-related text from the manuscript and let us know how you would like to update your Funding Statement. Currently, your Funding Statement reads as follows:

“The present project was supported by the King Gustav V Jubilee Clinical Cancer Research Foundation, Gothenburg, Sweden, and the Western County Council Clinical Research Foundation [VGFOUREG-374501], Sahlgrenska Academy, Gothenburg, Sweden.”

Please include your amended statements within your cover letter; we will change the online submission form on your behalf.

4. Please upload a new copy of Figures 1,2, 3, 4 and 5 as the detail is not clear. Please follow the link for more information:  https://journals.plos.org/plosone/s/figures

5. If the reviewer comments include a recommendation to cite specific previously published works, please review and evaluate these publications to determine whether they are relevant and should be cited. There is no requirement to cite these works unless the editor has indicated otherwise.

Additional Editor Comment:

Though the study is interesting to some extent but it requires a major revision before it can be considered for publication.

Major Comments

The study demonstrates that HBOT reverses radiation-induced downregulation of TLR5, TRIF, NRF2, HIF-α, and catalase, but the causal relationship between TLR5 signaling and antioxidant response remains unclear. Are the protective effects of HBOT mediated through TLR5? Co-treatment experiments with HBOT and TLR5 modulators (agonist/antagonist) would help establish this link.

The authors note that flagellin did not significantly alter antioxidant gene expression, yet it reduced cell death. This suggests TLR5-mediated protection may involve non-antioxidant pathways (NF-κB survival signaling). Further discussion or data on downstream effectors (Bcl-2, caspase activity) would add mechanistic insight.

Different HBOT schedules were used for HUVEC (two sessions) vs. HeLa/UROtsa (one session), justified by pilot data. However, the rationale for the 7-hour interval for HUVEC is not explained in depth. A brief description of pilot results in the main text or supplementary material would improve transparency.

The dose and timing of HBOT (200 kPa for 90 min, 3–10 h post-irradiation) are consistent with clinical protocols, but a discussion of how these parameters translate to in vivo settings would enhance clinical relevance.

The use of TH1020 as a TLR5 antagonist is appropriate, but its selectivity and efficacy in HeLa cells should be validated or referenced. Is there evidence that TH1020 blocks flagellin-induced signaling in this cell line?

The finding that TLR5 and TRIF expression increased with combined flagellin + TH1020 treatment is interesting but speculative. Could this represent compensatory feedback or off-target effects? Additional controls would strengthen the conclusion.

The study relies on mRNA expression of antioxidants (SOD, catalase) as proxies for oxidative stress. Direct measurement of ROS (e.g., DCFDA assay) or oxidative damage markers (8-OHdG, protein carbonyls) would provide more direct evidence of redox changes after HBOT and radiation.

Error bars in figures are described as SEM, but the number of replicates per group is not always clear (Fig 1a: n=2–7). Please specify exact n values for each condition in figure legends.

For multiple comparisons, the use of non-parametric tests is justified, but post-hoc adjustment methods should be explicitly stated in the results section.

Minor

Introduction could better highlight the novelty of combining HBOT and TLR5 modulation in epithelial radioprotection, as opposed to prior work in immune cells or animal models.

Ensure all figure labels are legible at publication size. Fig 1c microphotographs would benefit from scale bars and higher contrast.

The statement “additional data can be available upon request” does not fully comply with PLOS ONE’s data policy. Consider depositing key datasets in a public repository (e.g., Figshare, Zenodo) and providing an accession number.

Some citations in the text (Ref 25 on RNA:DNA hybrids) are not clearly connected to the results. Please contextualize their relevance.

[Note: HTML markup is below. Please do not edit.]

Reviewers' comments:

Reviewer's Responses to Questions

**Comments to the Author**

1. Is the manuscript technically sound, and do the data support the conclusions?

Reviewer #1: Partly

**********

2. Has the statistical analysis been performed appropriately and rigorously?

Reviewer #1: I Don't Know

**********

3. Have the authors made all data underlying the findings in their manuscript fully available?

The PLOS Data policy

Reviewer #1: Yes

**********

4. Is the manuscript presented in an intelligible fashion and written in standard English?

Reviewer #1: Yes

**********

Reviewer #1: 1 the animal experiment need add to test radioprotective effects of hyperbaric

oxygen (HBOT)。

2 the graphabstract need draw to indicate the finding of this study highlight

3 what the mechanism of radioprotective�

4 HUVEC, UROtsa and HeLa cells, cultured in 96-well plates, were irradiated 2-20 Gy at a dose rate of 3.65 Gy/min �what the detailed dose to each cell�please point out directly,also the radiation dose will not kill the cell ?

**********

what does this mean?). If published, this will include your full peer review and any attached files.

If you choose “no”, your identity will remain anonymous but your review may still be made public.

**Do you want your identity to be public for this peer review?**  For information about this choice, including consent withdrawal, please see our Privacy Policy

Reviewer #1: No

**********

[NOTE: If reviewer comments were submitted as an attachment file, they will be attached to this email and accessible via the submission site. Please log into your account, locate the manuscript record, and check for the action link "View Attachments". If this link does not appear, there are no attachment files.]

To ensure your figures meet our technical requirements, please review our figure guidelines: https://journals.plos.org/plosone/s/figures

You may also use PLOS’s free figure tool, NAAS, to help you prepare publication quality figures: https://journals.plos.org/plosone/s/figures#loc-tools-for-figure-preparation.

NAAS will assess whether your figures meet our technical requirements by comparing each figure against our figure specifications.

# Author comment: Author response to Decision Letter 1

**DOI:** 10.1371/journal.pone.0356247.r002

30 Jan 2026

Gothenburg, Sweden, 29 January 2026

Dear Editor,

Thank you for the opportunity to revise and resubmit our manuscript. We have carefully addressed all comments and suggestions and have revised the manuscript as detailed below. The manuscript has also been updated to comply with PLOS ONE’s style requirements. The funding information was removed from the Acknowledgments section.

Daniel Giglio

MD, PhD, Associate Professor

Questions/comments addressed by the Editor and Reviewer

1) The study demonstrates that HBOT reverses radiation-induced downregulation of TLR5, TRIF, NRF2, HIF-α, and catalase, but the causal relationship between TLR5 signaling and antioxidant response remains unclear. Are the protective effects of HBOT mediated through TLR5? Co-treatment experiments with HBOT and TLR5 modulators (agonist/antagonist) would help establish this link.

Answer: We show that HBOT treatment reverses radiation-induced alterations in antioxidant defense pathways, including changes in catalase and related regulators (Figure 2). Radiation exposure also modulated TLR5 expression, and this effect was similarly reversed by HBOT. However, pharmacological modulation of TLR5 signaling—using either a TLR5 agonist or antagonist—did not significantly affect antioxidant responses (Supplementary Figure 1).

Taken together, these findings indicate that although HBOT normalizes both antioxidant markers and TLR5 expression following radiation, our data does not support a causal role for TLR5 signaling in mediating the antioxidant effects of HBOT. Consequently, the present study cannot conclude that the radioprotective effects of HBOT are directly mediated through TLR5. This limitation and its implications are discussed in the Discussion section (lines 260-278).

2) The authors note that flagellin did not significantly alter antioxidant gene expression, yet it reduced cell death. This suggests TLR5-mediated protection may involve non-antioxidant pathways (NF-κB survival signaling). Further discussion or data on downstream effectors (Bcl-2, caspase activity) would add mechanistic insight.

Answer: We have added new sections to the Discussion (lines 260-270) discussing the possibility that flagellin affects non-TLR5 pathways including the NAIP5-NLRC4 inflammasome and caspase-1 [1].

3) Different HBOT schedules were used for HUVEC (two sessions) vs. HeLa/UROtsa (one session), justified by pilot data. However, the rationale for the 7-hour interval for HUVEC is not explained in depth. A brief description of pilot results in the main text or supplementary material would improve transparency.

Answer: We have added the following sections in Methods ”For HUVEC, exposing irradiated cells 6 h later with HBOT cell death induced an increase in cell death compared with sham-treated irradiated cells. Therefore, another protocol for HUVEC was employed where HUVEC cells were exposed to HBOT twice (3 h and 10 h post-irradiation) after irradiation for HUVEC. The rationale for a seven-hour time window between sessions of HBOT was for the cells to recover after being outside of the incubator.”

4) The dose and timing of HBOT (200 kPa for 90 min, 3–10 h post-irradiation) are consistent with clinical protocols, but a discussion of how these parameters translate to in vivo settings would enhance clinical relevance.

Answer: We have added the following sentence “During HBOT, the cells were placed in a hyperbaric chamber (GDA, Gothenburg, Sweden) and exposed to 200 kPa for 90 minutes, conditions shown to be effective against radiation-induced side effects in animal models and clinical practice [2, 3].

5) The use of TH1020 as a TLR5 antagonist is appropriate, but its selectivity and efficacy in HeLa cells should be validated or referenced. Is there evidence that TH1020 blocks flagellin-induced signaling in this cell line?

Answer: We currently provide evidence for functional TLR5 in HeLA as has been reported previously by other research groups [4]. The radioprotective effects on proliferation by TLR5 were also totally reversed by TH1020 in irradiated cells. TH1020 had at the same time no effects on proliferation in control cells. No studies to our knowledge have studied effects of TH1020 on HeLa. However, TH1020 has been shown to inhibit TLR5 signaling pathway in multiple cellular contexts, including hepatocytes and HEK-hTLR5 cells, without apparent cytotoxicity [5, 6].

6) The finding that TLR5 and TRIF expression increased with combined flagellin + TH1020 treatment is interesting but speculative. Could this represent compensatory feedback or off-target effects? Additional controls would strengthen the conclusion.

Answer: We acknowledge that these findings were puzzling. Besides activating TLR5, flagellin may activate the NAIP5-NLRC4 inflammasome [1]. The focus of the study was TLR5, downstream mediators and any connection to antioxidation. Whether flagellin activates the NAIP5-NLRC4 or connected pathways (caspase-1/ IL-1β) in HeLA were therefore not examined. We have discussed this further in Discussion (lines 260-270).

7) The study relies on mRNA expression of antioxidants (SOD, catalase) as proxies for oxidative stress. Direct measurement of ROS (e.g., DCFDA assay) or oxidative damage markers (8-OHdG, protein carbonyls) would provide more direct evidence of redox changes after HBOT and radiation.

Answer: We agree that direct measurements of ROS or oxidative damage markers could provide complementary evidence. However, our study focused on the transcriptional regulation of antioxidant defense pathways as an established proxy for redox changes, which is widely used in the field, as well as cell count. Importantly, the observed normalization of antioxidant mRNA levels after HBOT provides strong indirect support for a redox-modulating effect in response to radiation. We have clarified this limitation in the revised manuscript on lines 276-278.

8) Error bars in figures are described as SEM, but the number of replicates per group is not always clear (Fig 1a: n=2–7). Please specify exact n values for each condition in figure legends.

Answer: The figures in the manuscript have been adjusted as required, and the N-values are specified for each figure.

9) For multiple comparisons, the use of non-parametric tests is justified, but post-hoc adjustment methods should be explicitly stated in the results section.

Answer: We thank the reviewer for observing this. We have now added the information “Multiple comparisons were analyzed using the Kruskal-Wallis test followed by Dunn’s multiple comparison test” to the figures in the Result section.

10) Introduction could better highlight the novelty of combining HBOT and TLR5 modulation in epithelial radioprotection, as opposed to prior work in immune cells or animal models.

Answer: The primary aims of the present study were to investigate whether HBOT can reverse irradiation-induced changes, including modulation of TLR5 and antioxidant pathways, and to explore potential downstream pathways activated by TLR5. We have clarified this point in the revised Introduction in Aims (lines 70-76). While the direct combination of HBOT and TLR5 modulation has not yet been addressed, this represents a novel avenue that we plan to explore in future studies.

11) Ensure all figure labels are legible at publication size. Fig 1c microphotographs would benefit from scale bars and higher contrast.

Answer: We have revised Fig 1c and added a scale bar.

12) The statement “additional data can be available upon request” does not fully comply with PLOS ONE’s data policy. Consider depositing key datasets in a public repository (e.g., Figshare, Zenodo) and providing an accession number.

Answer: We have now added the complete data set as supplementary data.

13) Some citations in the text (Ref 25 on RNA:DNA hybrids) are not clearly connected to the results. Please contextualize their relevance.

Answer: We thank the reviewer for observing this. We have omitted the reference and the sentence in the revised version of the manuscript.

References

1. Tenthorey JL, Haloupek N, Lopez-Blanco JR, Grob P, Adamson E, Hartenian E, et al. The structural basis of flagellin detection by NAIP5: A strategy to limit pathogen immune evasion. Science. 2017;358(6365):888-93. doi: 10.1126/science.aao1140. PubMed PMID: 29146805; PubMed Central PMCID: PMCPMC5842810.

2. Oscarsson N, Muller B, Rosen A, Lodding P, Molne J, Giglio D, et al. Radiation-induced cystitis treated with hyperbaric oxygen therapy (RICH-ART): a randomised, controlled, phase 2-3 trial. Lancet Oncol. 2019;20(11):1602-14. Epub 20190916. doi: 10.1016/S1470-2045(19)30494-2. PubMed PMID: 31537473.

3. Oscarsson N, Ny L, Molne J, Lind F, Ricksten SE, Seeman-Lodding H, et al. Hyperbaric oxygen treatment reverses radiation induced pro-fibrotic and oxidative stress responses in a rat model. Free Radic Biol Med. 2017;103:248-55. Epub 20161227. doi: 10.1016/j.freeradbiomed.2016.12.036. PubMed PMID: 28034833.

4. Lim JS, Nguyen KC, Nguyen CT, Jang IS, Han JM, Fabian C, et al. Flagellin-dependent TLR5/caveolin-1 as a promising immune activator in immunosenescence. Aging Cell. 2015;14(5):907-15. Epub 20150730. doi: 10.1111/acel.12383. PubMed PMID: 26223660; PubMed Central PMCID: PMCPMC4568978.

5. Das N, Dewan V, Grace PM, Gunn RJ, Tamura R, Tzarum N, et al. HMGB1 Activates Proinflammatory Signaling via TLR5 Leading to Allodynia. Cell Rep. 2016;17(4):1128-40. doi: 10.1016/j.celrep.2016.09.076. PubMed PMID: 27760316; PubMed Central PMCID: PMCPMC5087801.

6. Bai H, Sun F, Yang G, Wang L, Zhang Q, Zhang Q, et al. CBLB502, a Toll-like receptor 5 agonist, offers protection against radiation-induced male reproductive system damage in mice. Biol Reprod. 2019;100(1):281-91. doi: 10.1093/biolre/ioy173. PubMed PMID: 30084935.

# Peer review: Decision Letter 1

**DOI:** 10.1371/journal.pone.0356247.r003

**Authors:** Budheswar Dehury

28 May 2026

Hyperbaric oxygen treatment and toll-like receptor 5 in radioprotection of epithelial cells

PLOS One

Dear Dr. Giglio,

Thank you for submitting your manuscript to PLOS ONE. After careful consideration, we feel that it has merit but does not fully meet PLOS ONE’s publication criteria as it currently stands. Therefore, we invite you to submit a revised version of the manuscript that addresses the points raised during the review process.

==============================

Both the reviewers have recommended revision, hence, I suggest you to revise your MS addressing each comment raised by the reviewers.

==============================

Please submit your revised manuscript by Jul 12 2026 11:59PM. If you will need more time than this to complete your revisions, please reply to this message or contact the journal office at plosone@plos.org. When you're ready to submit your revision, log on to https://www.editorialmanager.com/pone/ and select the 'Submissions Needing Revision' folder to locate your manuscript file.

- A letter that responds to each point raised by the academic editor and reviewer(s). You should upload this letter as a separate file labeled 'Response to Reviewers'.
- A marked-up copy of your manuscript that highlights changes made to the original version. You should upload this as a separate file labeled 'Revised Manuscript with Track Changes'.
- An unmarked version of your revised paper without tracked changes. You should upload this as a separate file labeled 'Manuscript'.

If you would like to make changes to your financial disclosure, please include your updated statement in your cover letter. Guidelines for resubmitting your figure files are available below the reviewer comments at the end of this letter.

If applicable, we recommend that you deposit your laboratory protocols in protocols.io to enhance the reproducibility of your results. Protocols.io assigns your protocol its own identifier (DOI) so that it can be cited independently in the future. For instructions see: https://journals.plos.org/plosone/s/submission-guidelines#loc-laboratory-protocols. Additionally, PLOS ONE offers an option for publishing peer-reviewed Lab Protocol articles, which describe protocols hosted on protocols.io. Read more information on sharing protocols at https://plos.org/protocols?utm_medium=editorial-email&utm_source=authorletters&utm_campaign=protocols.

As the corresponding author, your ORCID iD is verified in the submission system and will appear in the published article. PLOS supports the use of ORCID, and we encourage all coauthors to register for an ORCID iD and use it as well. Please encourage your coauthors to verify their ORCID iD within the submission system before final acceptance, as unverified ORCID iDs will not appear in the published article. *Only*  the individual author can complete the verification step; PLOS staff *cannot*  verify ORCID iDs on behalf of authors.

We look forward to receiving your revised manuscript.

Kind regards,

Budheswar Dehury

Academic Editor

PLOS One

Journal Requirements:

If the reviewer comments include a recommendation to cite specific previously published works, please review and evaluate these publications to determine whether they are relevant and should be cited. There is no requirement to cite these works unless the editor has indicated otherwise.

Additional Editor Comments:

Thank you for submitting your manuscript to PLOS ONE. After careful consideration, we feel that it has merit but does not fully meet PLOS ONE’s publication criteria as it currently stands. Therefore, we invite you to submit a revised version of the manuscript that addresses the points raised during the review process.

[Note: HTML markup is below. Please do not edit.]

Reviewers' comments:

Reviewer's Responses to Questions

**Comments to the Author**

Reviewer #1: All comments have been addressed

Reviewer #2: (No Response)

**********

2. Is the manuscript technically sound, and do the data support the conclusions?

Reviewer #1: Yes

Reviewer #2: Partly

**********

3. Has the statistical analysis been performed appropriately and rigorously?

Reviewer #1: Yes

Reviewer #2: Yes

**********

4. Have the authors made all data underlying the findings in their manuscript fully available?

The PLOS Data policy

Reviewer #1: No

Reviewer #2: Yes

**********

5. Is the manuscript presented in an intelligible fashion and written in standard English?

Reviewer #1: No

Reviewer #2: Yes

**********

Reviewer #1: why the sample size is 12 in the supplemantary data ?it need so many repulation ?please explain it

Reviewer #2: Sallam et al. and co-authors have nicely presented how HBOT can reverse radiation-induced alterations in inflammatory and antioxidant pathways. Though all the figures and supplementary data cover the story. I have couple of remarks that should be covered to finalize this manuscript.

1. Sallam et al. and co-authors have suggested a mechanistic role of HBOT in minimizing radiation-induced cellular toxicity and downstream inflammatory and oxidative pathways, but they focused only on the transcriptional regulation. In contrast to current study, Jia Liu et al. 2022 (PMID: 36495316) earlier demonstrated that HBOT co-exposure with radiation significantly strengthened cytotoxicity via ferroptotic cell death in oral squamous cell carcinoma cells. At this stage, it’s highly recommended to confirm this mechanism at protein level as well. Radiation induced toxicity and NRF2 mediated regulation of oxidative stress need to be confirmed by detecting ROS, 4-HNE or MDA.

2. Sallam et al. and co-authors have demonstrated that HBOT regulates TLR5 connected downstream signaling. While Rinaldi et al. 2011 and Kang et al. 2015 (PMID: 21567111 and 25973000) have highlighted the link between HBOT and other TLRs (TLR2 and TLR4), it's also advisable to check the levels of other TLRs in connection with radiation.

**********

what does this mean?). If published, this will include your full peer review and any attached files.

If you choose “no”, your identity will remain anonymous but your review may still be made public.

**Do you want your identity to be public for this peer review?**  For information about this choice, including consent withdrawal, please see our Privacy Policy

Reviewer #1: No

Reviewer #2: No

**********

[NOTE: If reviewer comments were submitted as an attachment file, they will be attached to this email and accessible via the submission site. Please log into your account, locate the manuscript record, and check for the action link "View Attachments". If this link does not appear, there are no attachment files.]

To ensure your figures meet our technical requirements, please review our figure guidelines: https://journals.plos.org/plosone/s/figures

You may also use PLOS’s free figure tool, NAAS, to help you prepare publication quality figures: https://journals.plos.org/plosone/s/figures#loc-tools-for-figure-preparation.

NAAS will assess whether your figures meet our technical requirements by comparing each figure against our figure specifications.

# Author comment: Author response to Decision Letter 2

**DOI:** 10.1371/journal.pone.0356247.r004

29 May 2026

Gothenburg, Sweden, 28 May 2026

Dear Editor,

Thank you for the opportunity to revise and resubmit our manuscript. We have carefully addressed all comments and suggestions. No further changes have been made in the manuscript.

Daniel Giglio

MD, PhD, Associate Professor

Response to reviewers´comments

Reviewer #1: why the sample size is 12 in the supplemantary data ?it need so many repulation ?please explain it

Answer: When we made multiple comparisons with Kruskal-Wallis for qPCR data we chose to have n=12 to have a sufficient number of samples to compensate for variations between samples.

Reviewer #2:

1. Sallam et al. and co-authors have suggested a mechanistic role of HBOT in minimizing radiation-induced cellular toxicity and downstream inflammatory and oxidative pathways, but they focused only on the transcriptional regulation. In contrast to current study, Jia Liu et al. 2022 (PMID: 36495316) earlier demonstrated that HBOT co-exposure with radiation significantly strengthened cytotoxicity via ferroptotic cell death in oral squamous cell carcinoma cells. At this stage, it’s highly recommended to confirm this mechanism at protein level as well. Radiation induced toxicity and NRF2 mediated regulation of oxidative stress need to be confirmed by detecting ROS, 4-HNE or MDA.

Answer: In the present study we assessed radiotherapy-induced toxicity and effects of HBO2 on several different important biological pathways primarily on the mRNA level. We have in Discussion in the section covering limitations highlighted that oxidative stress was only measured indirectly by measuring antioxidants.

2. Sallam et al. and co-authors have demonstrated that HBOT regulates TLR5 connected downstream signaling. While Rinaldi et al. 2011 and Kang et al. 2015 (PMID: 21567111 and 25973000) have highlighted the link between HBOT and other TLRs (TLR2 and TLR4), it's also advisable to check the levels of other TLRs in connection with radiation.

Answer: We acknowledge that other TLRs may be affected by irradiation as well. We have for example observed that radiation may downregulate TLR4 in the rat urinary bladder (1). However, based on our previous animal model of radiation-induced cervicitis, we observed only that TLR5 increased in expression among all TLRs (2). Moreover, the role of TLR5 has also been demonstrated in other models of radiation-induced radiotoxicity (3, 4). The article referred to by Reviewer 2 by Rinaldi et. al. (2011) describes down-regulation of TLR2 and TLR4 not in radiation-induced toxicity but in zymosan-induced general inflammation and the effects by HBO2 (5). The other article referred to by Reviewer 2 by Kang et. al. (2015) deals with spinal cord injury (and not radiation) and activation of TLR4 and effects of HBO2 (6).

References

1. Giglio D, Wasen C, Molne J, Suchy D, Swanpalmer J, Jabonero Valbuena J, et al. Downregulation of toll-like receptor 4 and IL-6 following irradiation of the rat urinary bladder. Clin Exp Pharmacol Physiol. 2016;43(7):698-705.

2. Mukanyangezi MF, Podmolikova L, Al Hydad W, Tobin G, Giglio D. Radiation induces changes in toll-like receptors of the uterine cervix of the rat. PLoS One. 2019;14(4):e0215250.

3. Burdelya LG, Krivokrysenko VI, Tallant TC, Strom E, Gleiberman AS, Gupta D, et al. An agonist of toll-like receptor 5 has radioprotective activity in mouse and primate models. Science. 2008;320(5873):226-30.

4. Wang Q, Duan J, Hong J, Ding K, Tai F, Zhu J, et al. Toll-like Receptor Agonist CBLB502 Protects Against Radiation-induced Intestinal Injury in Mice. In Vivo. 2024;38(4):1636-48.

5. Rinaldi B, Cuzzocrea S, Donniacuo M, Capuano A, Di Palma D, Imperatore F, et al. Hyperbaric oxygen therapy reduces the toll-like receptor signaling pathway in multiple organ failures. Intensive Care Med. 2011;37(7):1110-9.

6. Kang N, Hai Y, Yang J, Liang F, Gao CJ. Hyperbaric oxygen intervention reduces secondary spinal cord injury in rats via regulation of HMGB1/TLR4/NF-kappaB signaling pathway. Int J Clin Exp Pathol. 2015;8(2):1141-53.

Attachment Submitted filename: PlosOne response to reviewers 28 May 2026.pdf

# Peer review: Decision Letter 2

**DOI:** 10.1371/journal.pone.0356247.r005

**Authors:** Budheswar Dehury

2 Aug 2026

Hyperbaric oxygen treatment and toll-like receptor 5 in radioprotection of epithelial cells

PONE-D-25-51156R2

Dear Dr. Giglio,

We’re pleased to inform you that your manuscript has been judged scientifically suitable for publication and will be formally accepted for publication once it meets all outstanding technical requirements.

Within one week, you’ll receive an e-mail detailing the required amendments. When these have been addressed, you’ll receive a formal acceptance letter and your manuscript will be scheduled for publication.

An invoice will be generated when your article is formally accepted. Please note, if your institution has a publishing partnership with PLOS and your article meets the relevant criteria, all or part of your publication costs will be covered. Please make sure your user information is up-to-date by logging into Editorial Manager at Editorial Manager® and clicking the ‘Update My Information' link at the top of the page. For questions related to billing, please contact billing support.

If your institution or institutions have a press office, please notify them about your upcoming paper to help maximize its impact. If they’ll be preparing press materials, please inform our press team as soon as possible -- no later than 48 hours after receiving the formal acceptance. Your manuscript will remain under strict press embargo until 2 pm Eastern Time on the date of publication. For more information, please contact onepress@plos.org.

Kind regards,

Budheswar Dehury

Academic Editor

PLOS One

Additional Editor Comments (optional):

The authors have addressed most of the comments raised by the reviewers previously.

Reviewers' comments:

Reviewer's Responses to Questions

**Comments to the Author**

Reviewer #1: All comments have been addressed

Reviewer #2: All comments have been addressed

**********

2. Is the manuscript technically sound, and do the data support the conclusions?

Reviewer #1: Yes

Reviewer #2: Yes

**********

3. Has the statistical analysis been performed appropriately and rigorously?

Reviewer #1: Yes

Reviewer #2: Yes

**********

4. Have the authors made all data underlying the findings in their manuscript fully available?

The PLOS Data policy

Reviewer #1: (No Response)

Reviewer #2: Yes

**********

5. Is the manuscript presented in an intelligible fashion and written in standard English?

Reviewer #1: (No Response)

Reviewer #2: Yes

**********

Reviewer #1: (No Response)

Reviewer #2: Authors have responded to the points that I raised. Hopefully, addition of other TLRs interaction in radioprotection will provide more weightage to your study.

**********

what does this mean?). If published, this will include your full peer review and any attached files.

If you choose “no”, your identity will remain anonymous but your review may still be made public.

**Do you want your identity to be public for this peer review?**  For information about this choice, including consent withdrawal, please see our Privacy Policy

Reviewer #1: No

Reviewer #2: No

**********

# Editor report: Acceptance letter

**DOI:** 10.1371/journal.pone.0356247.r006

**Authors:** Budheswar Dehury

PONE-D-25-51156R2

PLOS One

Dear Dr. Giglio,

I'm pleased to inform you that your manuscript has been deemed suitable for publication in PLOS One. Congratulations! Your manuscript is now being handed over to our production team.

At this stage, our production department will prepare your paper for publication. This includes ensuring the following:

* All references, tables, and figures are properly cited

* All relevant supporting information is included in the manuscript submission,

* There are no issues that prevent the paper from being properly typeset

You will receive further instructions from the production team, including instructions on how to review your proof when it is ready. Please keep in mind that we are working through a large volume of accepted articles, so please give us a few days to review your paper and let you know the next and final steps.

Lastly, if your institution or institutions have a press office, please let them know about your upcoming paper now to help maximize its impact. If they'll be preparing press materials, please inform our press team within the next 48 hours. Your manuscript will remain under strict press embargo until 2 pm Eastern Time on the date of publication. For more information, please contact onepress@plos.org.

You will receive an invoice from PLOS for your publication fee after your manuscript has reached the completed accept phase. If you receive an email requesting payment before acceptance or for any other service, this may be a phishing scheme. Learn how to identify phishing emails and protect your accounts at https://explore.plos.org/phishing.

If we can help with anything else, please email us at customercare@plos.org.

Thank you for submitting your work to PLOS ONE and supporting open access.

Kind regards,

PLOS ONE Editorial Office Staff

on behalf of

Dr. Budheswar Dehury

Academic Editor

PLOS One
